# Awareness and practices of community pharmacists toward dextromethorphan misuse in Jeddah, Saudi Arabia: implications for regulation and public health

**DOI:** 10.3389/fmed.2025.1693207

**Published:** 2025-11-26

**Authors:** Aisha T. Alnami, Anfal S. Aljahdali, Haifa M. Alqahtani, Maha M. Khayat, Amal J. Aloshayni, Manal M. Aleesi, Mohammed M. Aldurdunji

**Affiliations:** 1 Department of Pharmaceutical Chemistry, Faculty of Pharmacy, King Abdulaziz University, Jeddah, Saudi Arabia; 2 Vaccines and Immunotherapy Unit, King Fahd Medical Research Center, King Abdulaziz University, Jeddah, Saudi Arabia; 3 Pharmaceutical Practices Department, College of Pharmacy, Umm Al-Qura University, Makkah, Saudi Arabia

**Keywords:** dextromethorphan, over-the-counter medicines, community pharmacists, substance misuse, Saudi Arabia, public health

## Abstract

**Background:**

Dextromethorphan (DXM) is an over-the-counter cough suppressant with recognized potential for misuse and abuse when taken at supratherapeutic doses. Community pharmacists are well positioned to identify and prevent such misuse, yet limited data exist from Saudi Arabia regarding their awareness, dispensing practices, and views on regulation.

**Methods:**

A cross-sectional survey was conducted among community pharmacists in Jeddah, Saudi Arabia, using a self-administered questionnaire. Descriptive statistics were used to summarize responses, and logistic regression was applied to identify factors associated with perceived increases in DXM sales and the adoption of restriction strategies.

**Results:**

A total of 252 pharmacists participated. Most respondents recognized DXM misuse as a local problem (206; 81.8%) and believed sales had increased over the preceding 2 years (131; 52.0%). Peer influence and social media promotion were identified as common drivers of misuse (206; 81.8%). The majority reported adopting restrictive dispensing practices, including refusal to sell to suspected abusers (178; 70.6%), and expressed strong support for tighter legal controls (230; 91.3%) and public awareness campaigns (198; 78.6%). Logistic regression showed that requests for non-OTC DXM products were associated with perceived increases in sales (OR = 2.13; 95% CI = 1.18–3.85; *p* = 0.012), while pharmacy location and previous dispensing behaviors predicted restrictive practices (OR = 1.96; 95% CI = 1.08–3.54; *p* < 0.05).

**Conclusion:**

Community pharmacists in Jeddah demonstrated awareness of DXM misuse and recognized the role of social and digital influences in promoting it. Their broad support for regulatory restrictions and preventive campaigns indicates readiness to contribute to public health strategies. Nonetheless, limited confidence in managing suspected cases highlights the need for targeted training and continued research across multiple regions to inform effective regulatory and educational responses.

## Introduction

Over-the-counter (OTC) medicines have been recognized as a public health concern for the past two decades, although they are generally considered safe when used within licensed therapeutic dose ranges (1, 2). A number of commonly available OTC medicines, including antihistamines, cough suppressants, codeine-containing preparations, analgesics, hypnotics, laxatives, and decongestants, contain active ingredients with abuse potential when consumed in higher-than-recommended doses (3–5). Such misuse and abuse can result in dependence and addiction, raising significant safety concerns (6, 7).

Dextromethorphan (DXM) is among the most prominent examples of over-the-counter (OTC) drugs with a well-documented potential for misuse and abuse (8). Reports from the American Association of Poison Control Centers showed a 300% increase in cases of DXM abuse among adolescents between 2000 and 2003 (9). Similarly, the United States National Survey on Drug Use and Health indicated that around 3.1 million people aged 12–25 years had used DXM-containing cough and cold medicines recreationally (10). Misuse has also been documented in other countries, including India, Japan, and China, with Sweden reclassifying DXM as a prescription-only drug in 1986 following the deaths of two adolescents (8, 9). In the United States, legal restrictions have been introduced to prevent sales of DXM products to individuals under 18 years of age, supported by the Food and Drug Administration (FDA) and the Consumer Healthcare Products Association (CHPA) (11, 12). In Saudi Arabia, the Saudi Food and Drug Authority (SFDA) classifies most DXM-containing products as prescription-only; however, some formulations, such as KAFOSED®, remain available over the counter, creating opportunities for misuse. Pharmacists continue to encounter OTC requests, particularly among younger individuals, prompting the SFDA to strengthen dispensing oversight and promote pharmacist education (13, 14). Community pharmacists thus play a critical role in monitoring non-medical DXM use and ensuring compliance with national regulations, consistent with broader public-health priorities under Saudi Vision 2030 (15, 16).

Pharmacologically, DXM is a synthetic analog of levorphanol and a non-opioid derivative of morphine (17). At therapeutic doses, it exerts antitussive activity primarily through sigma-1 receptor stimulation and inhibition of N-methyl-D-aspartate (NMDA) receptors (18). When taken at supratherapeutic doses, it additionally antagonizes NMDA, opioid, and α4β2 nicotinic receptors while inhibiting serotonin reuptake, leading to a broad spectrum of neurobehavioral effects including agitation, ataxia, nausea, vomiting, nystagmus, and speech disturbances (19–21). Psychological disturbances such as confusion, hallucinations, paranoia, and euphoria have also been reported (22). In severe cases, high-dose DXM ingestion has been linked to mania, violent behavior, and self-harm (23). In contrast, therapeutic use is usually associated only with mild side effects, including dizziness, drowsiness, and gastrointestinal upset (2, 24).

The increasing misuse of DXM has been attributed to its accessibility and the influence of peers and social media in promoting its euphoric effects (24, 25). Despite evidence of rising misuse globally, there are currently no published statistics or national studies describing DXM abuse in Saudi Arabia. This absence of local evidence highlights the need to investigate the extent of the problem and the role of healthcare professionals in mitigating it.

Community pharmacists are uniquely positioned to detect and respond to the misuse of OTC medicines. Their role extends beyond dispensing to include monitoring purchasing patterns, refusing inappropriate sales, providing patient education, and reporting suspected cases of abuse (26). Evaluating their knowledge, perceptions, and practices is therefore essential for guiding regulatory action, designing awareness campaigns, and informing pharmacist training programs.

This study aimed to evaluate the knowledge, perceptions, and practices of community pharmacists in Jeddah, Saudi Arabia, regarding the misuse and abuse of DXM. It specifically sought to examine pharmacists’ views on the prevalence of misuse, their confidence in managing suspected cases, and the strategies they employed to restrict access to DXM. Furthermore, it aimed to identify factors associated with changes in DXM sales and the implementation of restriction strategies through logistic regression analysis.

## Methods

### Study design and setting

This was a cross-sectional study conducted among community pharmacists in Jeddah, Saudi Arabia. Data were collected over a 12-month period, from May 2022 to May 2023, to ensure adequate coverage of various pharmacy locations and work shifts across the city. The specified duration reflected the time required to reach the target sample rather than a longitudinal follow-up. Pharmacies were selected using a geographically stratified random sampling approach. Jeddah was divided into five main districts (northern, southern, eastern, western, and central), and pharmacies within each district were randomly approached to ensure broad geographic representation and minimize selection bias. This method balanced feasibility with representativeness. Participation was voluntary and anonymous, and no identifying information was collected to encourage honest responses and minimize social-desirability bias. The questionnaire used neutral and non-judgmental wording to further reduce potential response distortion.

### Study population and sample size

The study population comprised licensed community pharmacists working in private-sector pharmacies across Jeddah, Saudi Arabia; hospital-based and trainee pharmacists were excluded. According to the Saudi Ministry of Health’s 2022 Statistical Yearbook (27), approximately 3,228 pharmacists were employed in this sector. The minimum required sample size was calculated using the Raosoft® sample size calculator, assuming a 95% confidence level, a 5% margin of error, and a 50% response distribution, yielding a target of 250 participants. This target was achieved, with responses obtained from 252 pharmacists. A 90% confidence level was selected to balance statistical precision with feasibility, considering the total number of eligible pharmacists and anticipated participation rates.

### Questionnaire development

The structured questionnaire was developed following a review of relevant literature addressing pharmacists’ knowledge, perceptions, and practices related to DXM misuse and abuse in international settings (1, 5, 6), as well as studies from Saudi Arabia concerning the misuse of other drugs with abuse potential, such as pregabalin (7). The draft questionnaire was reviewed by three academic experts in pharmacy practice to ensure content validity and was piloted with a small group of 10 community pharmacists in Jeddah to confirm clarity and feasibility. As the survey was bilingual (English and Arabic), forward–backward translation was applied to maintain accuracy across both versions. The final instrument was bilingual (English and Arabic) and web-based. The first page of the survey provided information about the study and included a consent statement that had to be acknowledged before participation. One of the survey items assessed whether pharmacists had dispensed more than one bottle of DXM to a single patient at one time. Response options were categorized as Not at all (0 times per month), Sometimes (1–3 times per month), and Frequently (≥4 times per month). These cut-offs were selected to provide approximate quantitative meaning to the terms and to improve consistency in interpretation.

### Data collection

Pharmacies were randomly selected within each of Jeddah’s five main districts (northern, southern, eastern, western, and central), and pharmacists on duty were invited to complete the electronic questionnaire. Responses were collected anonymously, coded, and stored securely to ensure confidentiality and data integrity.

### Data analysis

Data were analyzed using Minitab version 21.1. Descriptive statistics were applied to summarize sociodemographic characteristics and survey responses. Logistic regression analyses were used to identify predictors of two outcomes: (1) the perceived increase in DXM sales over the previous 2 years and (2) the implementation of restriction strategies toward suspected misusers or abusers. Results were reported as odds ratios (ORs) with 95% confidence intervals (CIs), and statistical significance was set at *p* < 0.05.

### Ethical considerations

Ethical approval was obtained from the Research Ethics Committee of the Faculty of Pharmacy at King Abdulaziz University (Reference No. PH-1443-42, Date: 1/2/2022). The study was conducted in accordance with the Implementing Regulations of the Law of Ethics of Research on Living Creatures in the Kingdom of Saudi Arabia and with the principles of the Declaration of Helsinki. Participation was voluntary, and informed consent was obtained electronically from all pharmacists prior to enrollment. Confidentiality and anonymity were strictly maintained, as no personally identifiable information was collected and all responses were stored securely with access restricted to the research team.

## Results

### Demographics of the respondents

The demographic characteristics of the participating pharmacists are presented in Table 1. A total of 252 community pharmacists participated in the study. The majority were male (199; 79.0%), and 53 (21.0%) were female. Most respondents were aged 25–34 years (141; 56.0%), followed by those aged 35–44 years (64; 25.4%).

**Table 1:** Sociodemographic and professional characteristics of the community pharmacists (*n* = 252).

Items	Subgroups	Sample (*n* = 252)	Percentage (%)
Gender	Male	201	79.76
	Female	51	20.24
Age	20–30	117	46.43
	31–40	109	43.25
	41–50	21	8.33
	Over 50	5	1.98
Education level	Bachelor’s in pharmacy	151	59.92
	Doctor of pharmacy (PharmD)	85	33.73
	Postgraduate	16	6.35
Work experience	1–4 years	68	26.98
	5–10 years	85	33.73
	Less than 1 year	31	12.30
	More than 10 years	68	26.98
Work-shift time	Midday	58	23.02
	Morning	104	41.27
	Night	90	35.72
Pharmacy location	East	14	5.56
	Middle	40	15.87
	North	133	52.78
	South	52	20.63
	West	13	5.16
Pharmacy type	Inside shopping center	18	7.14
	Main street	158	62.70
	Neighborhood	76	30.16
Graduate from	Non-Saudi university	174	69.05
	Saudi university	78	30.95
Nationality	Non-Saudi	171	67.86
	Saudi	81	32.14

Regarding education, 191 pharmacists (75.8%) held a bachelor’s degree in pharmacy, and 53 (21.0%) had a Doctor of Pharmacy (PharmD) qualification. Nearly half of the participants (126; 50.0%) had 5–10 years of professional experience, while 70 (27.8%) reported less than 5 years.

Pharmacists were distributed across all areas of Jeddah, with 73 (29.0%) from the northern district, 55 (21.8%) from the central district, 47 (18.7%) from the southern district, 40 (15.9%) from the eastern district, and 37 (14.7%) from the western district. Most respondents (193; 76.6%) worked in independent pharmacies, and 59 (23.4%) were employed in chain pharmacies.

### Community pharmacists’ perceptions toward customer’s misuse and abuse of DXM

Community pharmacists’ perceptions of DXM misuse and abuse are summarized in Table 2. Most respondents recognized DXM misuse as a local problem, with 206 pharmacists (81.8%) agreeing or strongly agreeing that such behavior occurs in Jeddah. Similarly, 217 pharmacists (86.1%) indicated they could identify suspected misusers, although only 183 (72.6%) felt they had sufficient knowledge to manage such cases confidently.

**Table 2:** Community pharmacists’ perceptions of dextromethorphan (DXM) misuse and abuse in Jeddah, Saudi Arabia (*n* = 252).

Items	Subgroups	Sample (*n* = 252)	Percentage (%)
Belief that DXM is misused or abused in Jeddah	Strongly agree	88	35%
	Agree	118	47%
	Neutral	32	13%
	Disagree	14	6%
	Strongly disagree	0	0%
Able to identify suspected DXM misusers or abusers	Strongly agree	82	33%
	Agree	135	54%
	Neutral	29	12%
	Disagree	6	2%
	Strongly disagree	0	0%
Feels sufficiently knowledgeable to deal with suspected DXM misusers or abusers	Strongly agree	62	25%
	Agree	121	48%
	Neutral	49	19%
	Disagree	16	6.35%
	Strongly disagree	4	1.5%
Perceives suspected DXM misuser/abuser as a regular customer	Strongly agree	33	13%
	Agree	77	31%
	Neutral	64	25%
	Disagree	62	25%
	Strongly disagree	16	6%
Gender of suspected DXM misuser/abuser	Male	158	62.70%
	Female	94	37.30%
Belief that DXM misusers/abusers are aware of its addictive effects	Strongly agree	72	29%
	Agree	96	38%
	Neutral	43	17%
	Disagree	34	13%
	Strongly disagree	7	3%
Belief that DXM misusers/abusers are aware of its euphoric effects	Strongly agree	72	29%
	Agree	96	38%
	Neutral	43	17%
	Disagree	34	13%
	Strongly disagree	7	3%
Belief that DXM misuse is initiated by information from friends or social media	Strongly agree	76	30%
	Agree	130	52%
	Neutral	34	13%
	Disagree	11	4%
	Strongly disagree	1	0.4%
Dispensing more than one DXM bottle to the same patient	Frequently (≥4 times per month)	3	1%
	Sometimes (1–3 times per month)	90	36%
	Not at all (0 times per month)	159	63%

More than 110 pharmacists (43.6%) believed that misusers were regular customers, and 158 (62.7%) reported that they were predominantly male. A total of 168 (66.7%) agreed that misusers were aware of DXM’s addictive potential, and an equal number (168; 66.7%) believed misusers were conscious of its euphoric effects. Peer influence and social media exposure were cited by 206 pharmacists (81.8%) as key factors contributing to misuse, highlighting the role of informal information sources.

Regarding dispensing practices, 159 pharmacists (63.1%) reported never supplying more than one bottle of DXM to a single patient, 90 (35.7%) reported doing so occasionally, and only 3 (1.2%) did so frequently. These findings indicate widespread awareness of DXM misuse among community pharmacists, coupled with moderate confidence in handling such cases and recognition of social factors that drive misuse.

### The observed change in the DXM sales over the past two years (Model 1)

Perceptions of pharmacists regarding changes in DXM sales over the past two years were assessed using a five-point Likert scale and subsequently collapsed into three analytical categories to facilitate interpretation: increase in trend (strongly agree + agree), no change (neutral), and decrease in trend (disagree + strongly disagree). Among the 252 respondents, 151 (59.9 %) perceived an increase in DXM sales, 74 (29.4 %) reported no change, and 28 (11.1 %) perceived a decrease.

Pharmacists’ perceptions of changes in DXM sales over the previous two years were analyzed to identify factors influencing their observations. Requests for non-OTC DXM products without prescriptions were significantly associated with pharmacists’ perception of increased sales (OR = 2.13; 95% CI 1.18–3.85; *p* = 0.012). In contrast, demographic and professional characteristics, including sex, age, education level, nationality, pharmacy type, pharmacy location, years of experience, and graduation origin, were not significantly related to this outcome (*p* > 0.05).

The multivariate regression model, summarized in Table 3, confirmed that requests for non-OTC DXM products remained the only independent factor significantly associated with pharmacists’ perception of increased sales after adjustment for potential confounders. No other demographic or occupational variables retained statistical significance. Some estimated odds ratios showed wide confidence intervals because of small subgroup sizes, and these findings should therefore be interpreted with caution.

**Table 3:** Predictors of increased sales of dextromethorphan (DXM) products among community pharmacists in Jeddah, Saudi Arabia.

Variable	Reference category	Adjusted OR (95% CI)
Gender of pharmacists	Female	0.98 (0.34–2.83)
Age of pharmacist (years)	20–30	31–40 → 0.60 (0.25–1.47) 41–50 → 1.36 × 10^5^ (0.00–4.16 × 10^106^) >50 → 1.09 × 10^5^ (0.00–1.19 × 10^231^)
Education level	Bachelor’s in pharmacy	Doctor of Pharmacy → 0.95 (0.38–2.37) Postgraduate → 1.87 (0.22–15.88)
Work-shift time	Midday	Night → 0.63 (0.20–1.96) Morning → 0.81 (0.26–2.53)
Work experience	<1 year	1–4 → 0.39 (0.04–3.69) 5–10 → 0.42 (0.04–3.63) >10 → 0.39 (0.04–3.41)
Pharmacy location	North Jeddah	East Jeddah → 3.6 × 10^5^ (0.00–2.40 × 10^253^) Middle Jeddah → 0.75 (0.26–2.18) South Jeddah → 1.95 (0.56–6.85)
Pharmacy type	Neighborhood	1.29 (0.14–11.95) Main street → 0.75 (0.28–1.97) Inside shopping center → 0.65 (0.13–3.30)
Graduate from	Non-Saudi university	Saudi university → 1.04 (0.65–2.68)
Age of abusers (years)	15–24	25–35 → 1.24 (0.31–5.01) >35 → 1.43 (0.20–10.39)

OR, odds ratio; CI, confidence interval.

### Strategies to limit the access of suspected abusers or misusers to DXM (Model 2)

Pharmacists’ responses to suspected dextromethorphan (DXM) misuse were examined to identify factors associated with adopting restrictive dispensing practices. Among the 252 respondents, 178 (70.6 %) reported refusing to sell DXM to suspected misusers, 60 (23.8 %) dispensed a smaller quantity or provided the product only after additional counselling, and 14 (5.6 %) continued dispensing as usual. Most participants expressed strong support for broader control measures, with 230 (91.3 %) agreeing that DXM should be placed under formal legal restriction and 198 (78.6 %) supporting its inclusion in public-awareness and anti-drug campaigns. Some of the factors examined are presented in Table 4, whereas others were discussed in the text.

**Table 4:** Reported strategies and predictors of restrictive practices to limit access to dextromethorphan (DXM) among community pharmacists in Jeddah, Saudi Arabia.

Variable	Reference category	Adjusted OR (95% CI)
Gender	Female	0.71 (0.24–2.07)
Age of pharmacist (years)	20–30	31–40 → 0.69 (0.27–1.81) 41–50 → 0.43 (0.08–2.20) >50 → 0.14 (0.01–4.19)
Education level	Bachelor’s in pharmacy	Doctor of Pharmacy → 5.39 (0.38–9.84) Postgraduate → 5.04 (1.39–7.94)^†^
Work-shift time	Midday	Night → 4.27 (0.11–4.03) Morning → 2.39 (0.34–4.90)
Work experience (years)	<1	1–4 → 0.24 (0.08–0.71)^†^ 5–10 → 0.22 (0.06–0.87)^†^ >10 → 0.18 (0.00–10.28)
Pharmacy type	Neighborhood	Main street → 0.49 (0.04–0.40)^†^ Inside shopping center → 0.20 (0.04–0.94)^†^
Gender of abusers	Female	0.39 (0.03–4.10)
Age of abusers (years)	15–24	25–35 → 1.39 (0.12–5.53) >35 → 0.84 (0.07–0.56)

OR, odds ratio; CI, confidence interval. ^†^indicates 95% CI excluding 1.0 (statistically significant association).

Pharmacists’ adoption of restrictive dispensing strategies was further analyzed using the second multivariate logistic regression model, with the results summarized in Table 4. Requests for non-OTC DXM products and pharmacy location were significantly associated with restrictive behaviors (OR = 1.96; 95% CI 1.08–3.54; *p* < 0.05). Education level, work experience, and pharmacy type also demonstrated significant associations. Pharmacists with postgraduate qualifications were more likely to implement restriction strategies than those with only a bachelor’s degree (adjusted OR = 5.04; 95% CI 1.39–7.94; *p* < 0.05). Conversely, those with 1–4 years or 5–10 years of experience were less likely to apply restrictions compared with pharmacists having < 1 year of experience (OR = 0.24; 95% CI 0.08–0.71 and OR = 0.22; 95% CI 0.06–0.87, respectively). Pharmacists working in main-street or shopping-center locations were also less likely to report restrictive practices than those in neighborhood pharmacies (OR = 0.49; 95% CI 0.04–0.40 and OR = 0.20; 95% CI 0.04–0.94, respectively).

Other demographic or occupational factors were not significantly associated with this outcome. Several odds ratios exhibited wide confidence intervals due to small subgroup sizes, and these findings should therefore be interpreted with caution.

## Discussion

This study explored community pharmacists’ perceptions, knowledge, and practices regarding dextromethorphan (DXM) misuse in Jeddah, Saudi Arabia. The findings showed that a substantial proportion of pharmacists recognized DXM misuse within their communities, with 206 pharmacists (81.8%) acknowledging it as a local problem and 217 (86.1%) indicating that they could identify suspected misusers. More than half of the respondents (131; 52.0%) believed that DXM sales had increased in recent years, and the majority cited peer influence and social media exposure (206; 81.8%) as key drivers of misuse. Logistic regression analysis identified that requests for non-OTC DXM products without prescription were significantly associated with pharmacists’ perception of increased DXM sales (OR = 2.13, 95% CI = 1.18–3.85, *p* = 0.012). Many pharmacists reported adopting restriction strategies such as refusing to sell DXM to suspected abusers (178; 70.6%) or limiting the dispensed quantity (60; 23.8%), while almost all supported tighter regulation (230; 91.3%) and inclusion of DXM in public-awareness efforts (198; 78.6%). Despite this vigilance, 69 pharmacists (27.4%) felt inadequately prepared to manage suspected misuse, indicating potential gaps in training and resource availability.

The recognition of DXM misuse by pharmacists in this study aligns with international observations. In Southern Europe, Perelló et al. reported that DXM was among the medicines most frequently associated with suspected abuse cases presenting in community pharmacies (28). Similar findings were described in Jordan, where Alabi et al. found that nearly all pharmacists surveyed had encountered DXM misuse in their communities (29). Within Saudi Arabia, Mobrad et al. also observed broad awareness of medicine misuse but noted limited pharmacist confidence in managing such cases (30). Comparable patterns were reported in the United Kingdom, where Cooper described pharmacists’ uncertainty and hesitation when faced with suspected OTC misuse, often linked to insufficient formal training (31). Collectively, these studies, together with the present findings, demonstrate that community pharmacists across different countries consistently recognize DXM misuse as a growing public-health issue, yet many continue to feel underprepared to intervene effectively.

The perception of increasing DXM sales reported by pharmacists in this study (131; 52.0%) may reflect broader international trends. Although actual sales data were not collected, this perception was significantly associated with receiving requests for non-OTC DXM products without prescription (OR = 2.13; 95% CI = 1.18–3.85; *p* = 0.012), suggesting that pharmacists’ observations could be linked to non-therapeutic demand. Comparable upward trends in recreational DXM use have been documented internationally. In Canada, a national survey of secondary school students reported that approximately one in 10 admitted to recreational DXM use (32). In the United States, emergency department surveillance identified a sharp rise in DXM abuse-related presentations among adolescents during the early 2000s (33). Similarly, European community pharmacy data have documented DXM-related misuse, with cases ranging from psychosomatic complaints to acute intoxication (28). The consistency between these international findings and local pharmacists’ perceptions may indicate that DXM misuse in Saudi Arabia reflects a parallel global pattern of recreational consumption.

The influence of social media and peer interaction was also emphasized by participating pharmacists, with 206 (81.8%) identifying these factors as key contributors to misuse. This perception is supported by studies describing how online platforms can normalize or trivialize the misuse of medicines such as DXM. Reports of “robo-tripping” as an adolescent trend illustrate how digital media can amplify risky behaviors (34), while the “Benadryl challenge” on TikTok underscores the dangers of viral social-media-driven drug experimentation (35). Social networks may further reinforce misperceptions about substance use: Cox et al. found that young adults frequently overestimated peer substance use, which was associated with greater personal risk-taking (36), and Strowger and Braitman observed that exposure to substance-related content on social media correlated with higher levels of actual substance use (37). Although the present study cannot establish causality, these converging findings support the pharmacists’ view that peer influence and digital exposure contribute to emerging patterns of DXM misuse.

The strategies reported by pharmacists to limit access to suspected DXM misusers, such as refusing to dispense (178; 70.6%) or restricting the quantity supplied (60; 23.8%), are broadly consistent with regulatory approaches adopted in other regions. Logistic regression analysis showed that pharmacy location and previous experience dispensing multiple bottles to a single customer were significant predictors of implementing restriction strategies (OR = 1.96; 95% CI = 1.08–3.54; *p* < 0.05). In the United States, several states have implemented age restrictions on DXM purchases, typically limiting sales to individuals aged 18 years or older (8). Across Europe, regulatory responses have included the reclassification of DXM as a prescription-only medicine in some countries, and the introduction of tamper-evident packaging in France to curb misuse (38). In Asia, Malaysia has classified DXM as a scheduled substance following increasing reports of non-medical use (39). Although regulatory frameworks differ, the alignment of local pharmacists’ restrictive dispensing behaviors with these formal international measures suggests that professional awareness and informal self-regulation may arise from shared recognition of DXM’s misuse potential.

Training and education also emerged as important considerations in this study, as 69 pharmacists (27.4%) reported feeling inadequately prepared to manage suspected misuse cases. Similar challenges have been documented internationally. In Poland, Pietrusiewicz et al. found that pharmacists’ counseling practices for common cold treatments were influenced by insufficient training and time constraints (40). In Qatar, simulated-client research indicated gaps in pharmacists’ ability to provide evidence-based advice for respiratory conditions (41). Likewise, in Indonesia, Mizranita and Nisa identified limited professional development opportunities as a barrier to effective management of minor ailments in community settings (42). These findings parallel those from the present study, suggesting that limited preparedness among community pharmacists is a recurrent global concern. Several authors have emphasized the need for structured continuing education and standardized practice guidelines to strengthen pharmacists’ ability to identify, counsel, and manage patients at risk of medicine misuse, including DXM (43). In Saudi Arabia, these insights may also guide the Saudi Food and Drug Authority in refining DXM dispensing regulations and inform the design of continuing-education initiatives that enhance pharmacists’ capacity to recognize and manage suspected misuse cases.

The public health implications of DXM misuse are noteworthy yet warrant cautious interpretation. Misuse has been most frequently reported among adolescents and young adults in many countries, groups that may be particularly influenced by social factors and misconceptions about the safety of over-the-counter medicines. In this study, pharmacists overwhelmingly supported preventive initiatives, with 198 respondents (78.6%) endorsing the inclusion of DXM in public awareness and anti-drug campaigns. Similar recommendations have been reported internationally, where targeted educational programs for youth have been compared to opioid awareness interventions that achieved measurable improvements in public understanding and reductions in non-medical use (44). The present findings highlight pharmacists’ readiness to contribute to community-based prevention strategies aimed at reducing DXM misuse.

This study has several limitations. First, it relied on self-reported data from pharmacists, which may be subject to recall and social desirability bias. Objective data sources, such as pharmacy sales or dispensing records, would provide a more accurate assessment of DXM distribution trends. Second, the study was confined to Jeddah, and results may not represent pharmacists’ perceptions in other Saudi regions or internationally. However, Jeddah’s diverse population, high pharmacy density, and consistent oversight under Saudi Food and Drug Authority regulations make it broadly comparable to other major Saudi cities. Therefore, the findings may cautiously be extrapolated to similar urban contexts, while differences in population characteristics and healthcare access in smaller or rural areas may limit generalizability. Third, the voluntary participation and online survey format may have introduced selection bias, favoring respondents with a greater professional interest in misuse-related topics. Finally, some regression analyses produced wide confidence intervals due to small subgroup sizes, and no adjustments were made for multiple testing; therefore, these statistical findings should be interpreted with caution.

Future research opportunities are evident. Expanding data collection to multiple regions across Saudi Arabia would allow for comparisons between urban and rural settings and support the development of a national overview of DXM misuse. Longitudinal and mixed-methods designs could help monitor trends over time and evaluate the effects of forthcoming regulatory or educational interventions. Exploring patient perspectives, particularly among adolescents and young adults, would complement pharmacists’ views and provide deeper insight into the behavioral and social determinants of misuse. Interventional studies, including pharmacist-focused training programs and public awareness campaigns, may also help determine the effectiveness of strategies designed to promote the safe and appropriate use of DXM-containing products.

## Conclusion

This study adds to the growing body of evidence on pharmacists’ perceptions of dextromethorphan (DXM) misuse by providing data from community pharmacists in Jeddah, Saudi Arabia. The findings indicate that most pharmacists recognized DXM misuse as a local concern (206; 81.8%), with more than half perceiving an increase in sales (131; 52.0%). Peer and social media influence were frequently identified as contributing factors (206; 81.8%), while a majority reported adopting restrictive dispensing strategies (178; 70.6%) and expressed strong support for tighter regulatory measures (230; 91.3%). However, limited confidence in managing suspected misuse (69; 27.4%) suggests that additional training and professional development are needed to strengthen pharmacists’ preparedness.

Although these results align with international reports, they remain limited by the self-reported design and single-city focus. Future studies incorporating larger, multi-regional samples and objective data sources, such as pharmacy sales records, would provide more comprehensive insight into DXM misuse patterns in Saudi Arabia. Initiatives aimed at enhancing pharmacists’ training, refining regulatory frameworks, and implementing targeted public health awareness programs may help mitigate the risks associated with DXM misuse and promote safer use of over-the-counter medications.

## Data Availability

The raw data supporting the conclusions of this article will be made available by the authors, without undue reservation.

## References

[1] Administration USF and D. Understanding over-the-counter medicines. (2018). Available online at: https://www.fda.gov/drugs/buying-using-medicine-safely/understanding-over-counter-medicines (Accessed November 1, 2025).

[2] Spangler DC Loyd CM Skor EE. Dextromethorphan: a case study on addressing abuse of a safe and effective drug. Subst Abuse Treat Prev Policy. (2016) 11:22. doi: 10.1186/s13011-016-0067-0 27333886 PMC4918034

[3] Sansgiry S Bhansali A Bapat S Xu Q. Abuse of over-the-counter medicines: a pharmacist’s perspective. Integr Pharm Res Pract. (2016) 6:1–6. doi: 10.2147/IPRP.S103494 29354545 PMC5774309

[4] Schifano F Chiappini S Miuli A Mosca A Santovito MC Corkery JM . Focus on over-the-counter drugs’ misuse: a systematic review on antihistamines, cough medicines, and decongestants. Front Psych. (2021) 12:657397. doi: 10.3389/fpsyt.2021.657397/full PMC8138162 34025478

[5] Abraham O Chmielinski J. Adolescents’ misuse of over-the-counter medications: the need for pharmacist-led intervention. Innov Pharm. (2018) 9:4. doi: 10.24926/iip.v9i3.979 PMC6302752 34007709

[6] Algarni M Hadi MA Yahyouche A Mahmood S Jalal Z. A mixed-methods systematic review of the prevalence, reasons, associated harms and risk-reduction interventions of over-the-counter (OTC) medicines misuse, abuse and dependence in adults. J Pharm Policy Pract. (2021) 14:76. doi: 10.1186/s40545-021-00350-7, PMID: 34517925 PMC8439034

[7] Alshahrani SM Orayj K Alqahtani AM Algahtany MA. Community pharmacists’ perceptions towards the misuse and abuse of pregabalin: a cross-sectional study from Aseer region, Saudi Arabia. Healthcare. (2021) 9:1281. doi: 10.3390/healthcare9101281, PMID: 34682961 PMC8535499

[8] Boyer E Burns J. Antitussives and substance abuse. Subst Abus Rehabil. (2013) 4:75. doi: 10.2147/SAR.S36761 PMC3931656 24648790

[9] Romanelli F Smith KM. Dextromethorphan abuse: clinical effects and management. J Am Pharm Assoc. (2009) 49:e20–7. doi: 10.1331/JAPhA.2009.08091, PMID: 19289333

[10] Lam SHF Homme J Avarello J Heins A Pauze D Mace S . Use of antitussive medications in acute cough in young children. JACEP Open. (2021) 2:e12467. doi: 10.1002/emp2.12467, PMID: 34179887 PMC8212563

[11] Storck M Black L Liddell M. Inhalant abuse and dextromethorphan. Child Adolesc Psychiatr Clin. (2016) 25:497–508. doi: 10.1016/j.chc.2016.03.007, PMID: 27338970

[12] Association CHP. CHPA applauds oregon lawmakers for age-18 sales law on cough medicine. (2017). Available online at: https://www.oregonlive.com/business/businesswire/2017/06/chpa_applauds_oregon_lawmakers.html (Accessed November 1, 2025).

[13] Almalki M FitzGerald G Clark M. Health care system in Saudi Arabia: an overview. East Mediterr Health J. (2011) 17:784–93. doi: 10.26719/2011.17.10.784, PMID: 22256414

[14] Alomi YA Alghamdi SJ Alattyh RA. National corporate pharmacy and therapeutic committee at the Ministry of Health, Saudi Arabia. Pharmacol Toxicol Biomed Rep. (2019) 4:24–7. doi: 10.5530/PTB.2018.4.9

[15] Khan TM Emeka PM Suleiman AK Alnutafy FS Aljadhey H. Pharmaceutical pricing policies and procedures in Saudi Arabia: a narrative review. Ther Innov Regul Sci. (2016) 50:236–40. doi: 10.1177/2168479015609648, PMID: 30227009

[16] Darraj A Almutairi M Alhassan O Aljammaz A Almansour I Alotaibi S . Attitudes and practices of physicians toward law enforcement on dispensing antibiotics without prescription antibiotics: findings from a cross-sectional survey. J Fam Med Prim Care. (2023) 12:679–85. doi: 10.4103/jfmpc.jfmpc_1942_22, PMID: 37312778 PMC10259565

[17] Information NC for B. PubChem compound summary for CID 5360696, dextromethorphan. (2022). Available online at: https://pubchem.ncbi.nlm.nih.gov/compound/Dextromethorphan (Accessed November 1, 2025).

[18] Nguyen L Thomas KL Lucke-Wold BP Cavendish JZ Crowe MS Matsumoto RR. Dextromethorphan: an update on its utility for neurological and neuropsychiatric disorders. Pharmacol Ther. (2016) 159:1–22. doi: 10.1016/j.pharmthera.2016.01.016, PMID: 26826604

[19] Shimozawa S Usuda D Sasaki T Tsuge S Sakurai R Kawai K . High doses of dextromethorphan induced shock and convulsions in a 19-year-old female: a case report. World J Clin Cases. (2023) 11:3870–6. doi: 10.12998/wjcc.v11.i16.3870, PMID: 37383112 PMC10294160

[20] Briggs SA Hall BJ Wells C Slade S Jaskowski P Morrison M . Dextromethorphan interactions with histaminergic and serotonergic treatments to reduce nicotine self-administration in rats. Pharmacol Biochem Behav. (2016) 142:1–7. doi: 10.1016/j.pbb.2015.12.004, PMID: 26704812 PMC4749472

[21] Chiang YC Ye LC Hsu KY Liao CW Hung TW Lo WJ . Beneficial effects of co-treatment with dextromethorphan on prenatally methadone-exposed offspring. J Biomed Sci. (2015) 22:19. doi: 10.1186/s12929-015-0126-2, PMID: 25890152 PMC4376496

[22] Dilich A Girgis C. Robo-tripping: a case of Robitussin abuse in a methadone maintenance patient. Psychosomatics. (2017) 58:544–50. doi: 10.1016/j.psym.2017.03.010, PMID: 28576306

[23] Administration D of JE. What is DXM?. (2020). Available online at: https://www.dea.gov/sites/default/files/2020-06/DXM-2020.pdf (Accessed November 1, 2025).

[24] Nawi AM Ismail R Ibrahim F Hassan MR Manaf MRA Amit N . Risk and protective factors of drug abuse among adolescents: a systematic review. BMC Public Health. (2021) 21:2088. doi: 10.1186/s12889-021-11906-2, PMID: 34774013 PMC8590764

[25] Bell LA Hadland SE. The changing landscape of adolescent and young adult substance use. J Adolesc Health. (2024) 75:706–8. doi: 10.1016/j.jadohealth.2024.07.024 39269379 PMC12004322

[26] Society RP. Pharmacy’s role in reducing harm and preventing drug deaths (Scotland). London: Royal Pharmaceutical Society (2021).

[27] Health SM of. Statistical yearbook. Riyadh: Ministry of Health (2023).

[28] Perelló M Rio-Aige K Guayta-Escolies R Gascón P Rius P Jambrina AM . Evaluation of medicine abuse trends in community pharmacies: the medicine abuse observatory (MAO) in a region of southern Europe. Int J Environ Res Public Health. (2021) 18:7818. doi: 10.3390/ijerph18157818, PMID: 34360110 PMC8345500

[29] Alabi KO Joshua F Akinwumi AI Adje DU Awodele O. Perception of community pharmacists on abuse of psychotropic medications among the consumers. Trop J Pharm Res. (2023) 21:2721–9. doi: 10.4314/tjpr.v21i12.30

[30] Mobrad AM Alghadeer S Syed W Al-Arifi MN Azher A Almetawazi MS . Knowledge, attitudes, and beliefs regarding drug abuse and misuse among community pharmacists in Saudi Arabia. Int J Environ Res Public Health. (2020) 17:1334. doi: 10.3390/ijerph17041334, PMID: 32092993 PMC7068280

[31] Cooper R. Surveillance and uncertainty: community pharmacy responses to over the counter medicine abuse. Health Soc Care Commun. (2013) 21:254–62. doi: 10.1111/hsc.12012, PMID: 23320510

[32] Antoniou T Juurlink DN. Dextromethorphan abuse. Can Med Assoc J. (2014) 186:E631–1. doi: 10.1503/cmaj.131676, PMID: 25135924 PMC4216279

[33] Finkelstein Y Goel G Hutson JR Armstrong J Baum CR Wax P . Drug misuse in adolescents presenting to the emergency department. Pediatr Emerg Care. (2017) 33:451–6. doi: 10.1097/PEC.0000000000000571, PMID: 26466148

[34] Linn KA Long MT Pagel PS. “Robo-tripping”: dextromethorphan abuse and its anesthetic implications. Anesthesiol Pain Med. (2014) 4:e20990. doi: 10.5812/aapm.20990, PMID: 25793175 PMC4358333

[35] Elkhazeen A Poulos C Zhang X Cavanaugh J Cain M. A TikTok ™ “Benadryl challenge” death—a case report and review of the literature. J Forensic Sci. (2023) 68:339–42. doi: 10.1111/1556-4029.15149 36173026

[36] Cox MJ DiBello AM Meisel MK Ott MQ Kenney SR Clark MA . Do misperceptions of peer drinking influence personal drinking behavior? Results from a complete social network of first-year college students. Psychol Addict Behav. (2019) 33:297–303. doi: 10.1037/adb0000455, PMID: 30869918 PMC6483870

[37] Strowger M Braitman AL. Using social network methodology to examine the effects of exposure to alcohol-related social media content on alcohol use: a critical review. Exp Clin Psychopharmacol. (2023) 31:280–93. doi: 10.1037/pha0000561 35357872 PMC10107381

[38] Jouanjus E Falcou A Deheul S Roussin A Lapeyre-Mestre M. Detecting the diverted use of psychoactive drugs by adolescents and young adults: a pilot study. Pharmacoepidemiol Drug Saf. (2018) 27:1286–92. doi: 10.1002/pds.4624, PMID: 30255533

[39] Roy AK Hsieh C Crapanzano K. Dextromethorphan addiction mediated through the NMDA system. J Addict Med. (2015) 9:499–501. doi: 10.1097/ADM.0000000000000152, PMID: 26441400

[40] Pietrusiewicz M Kopa-Stojak PN Pawliczak R. Pharmacist’s recommendations of over-the-counter treatments for the common cold - analysis of prospective cases in Poland. BMC Fam Pract. (2021) 22:216. doi: 10.1186/s12875-021-01561-2 34717562 PMC8556806

[41] Mohamed Ibrahim MI Awaisu A Palaian S Radoui A Atwa H. Do community pharmacists in Qatar manage acute respiratory conditions rationally? A simulated client study. J Pharm Health Serv Res. (2018) 9:33–9. doi: 10.1111/jphs.12204

[42] Mizranita V Nisa F. Perceptions of practice towards management of common ailments in Indonesian community pharmacies: a cross-sectional study. Int J Soc Health. (2024) 3:423–33. doi: 10.58860/ijsh.v3i7.216

[43] Alexa JM Bertsche T. An online cross-sectional survey of community pharmacists to assess information needs for evidence-based self-medication counselling. Int J Clin Pharm. (2023) 45:1452–63. doi: 10.1007/s11096-023-01624-7, PMID: 37532842 PMC10682211

[44] Pathak GN Chandy RJ Shah R Feldman SR. The pharmacist’s role in dermatology: patient medication adherence. J Dermatol. (2023) 50:1099–107. doi: 10.1111/1346-8138.16895, PMID: 37489577

